# Application of subtracted gDNA microarray-assisted Bulked Segregant Analysis for rapid discovery of molecular markers associated with day-neutrality in strawberry (*Fragaria x ananassa*)

**DOI:** 10.1038/srep32551

**Published:** 2016-09-02

**Authors:** Mian Chee Gor, Nitin Mantri, Edwin Pang

**Affiliations:** 1 School of Science, Health Innovations Research Institute, RMIT University, Melbourne, Victoria 3000, Australia

## Abstract

A Fragaria Discovery Panel (FDP; strawberry-specific SDA) containing 287 features was constructed by subtracting the pooled gDNA of nine non-angiosperm species from the pooled gDNA of five strawberry genotypes. This FDP was used for Bulk Segregant Analysis (BSA) to enable identification of molecular markers associated with day-neutrality. Analysis of hybridisation patterns of a short day (SD) DNA bulk and three day-neutral (DN) DNA bulks varying in flowering strength allowed identification of a novel feature, FaP2E11, closely linked to *CYTOKININ OXIDASE 1* (*CKX1*) gene possibly involved in promoting flowering under non-inductive condition. The signal intensities of FaP2E11 feature obtained from the strong DN bulk (DN1) is three fold higher than the short day bulk (SD), indicating that the putative marker may linked to a *CKX1* variant allele with lower enzyme activity. We propose a model for flowering regulation based on the hypothesis that flowering strength may be regulated by the copy number of FaP2E11-linked *CKX1* alleles. This study demonstrates the feasibility of the SDA-based BSA approach for the identification of molecular markers associated with day-neutrality in strawberry. This innovative strategy is an efficient and cost-effective approach for molecular marker discovery.

The advancement of DNA microarray facilitated genome characterisation of closely related individuals, enabling the detection of DNA variations and analysis of genomic diversity using simple experimental approaches^1^. We previously developed a novel Subtracted Diversity Array (SDA) technique by combining a modified Suppression Subtractive Hybridisation (SSH) technique with microarray for high throughput genotyping of angiosperm species. The prototype microarray involved subtraction of pooled genomic DNA between five non-angiosperm and 49 angiosperm species^2^. It has the capacity to correctly genotype plants used in the initial library construction up to family level, and plants not included during array development up to clade level^3^. We subsequently refined the SDA technique to efficiently eliminate common sequences between two DNA pools, and achieved a subtraction efficiency of 97.0%^4^. We also successfully extended the use of this technique for narrow subtraction at clade (Asterids-specific SDA) and genera (*Salvia*- and *Echinacea*-specific SDA) levels and achieved high subtraction efficiency between 88 to 99.6%^4^,^5^,^6^. The SDA technique is not only capable of fingerprinting a wide range of plants, but more importantly, identifying novel species and family-specific sequences, and markers associated with accumulation of bioactive compounds^4^,^5^,^6^. The SDA is also sensitive enough to detect 10% adulteration in dried herbal formulations^7^. However, this technique has never been employed for the discovery of molecular markers associated with important traits in polyploid crops, for instance, the octoploid strawberry (*Fragaria x ananassa*).

Strawberry is one of the most economically important soft fruits cultivated in the world^8^. They are highly favoured for their unique appearance, smell, taste and nutritional quality^9^,^10^. Development of elite strawberry cultivars with commercially important traits may be facilitated by use of molecular markers tightly linked to desired traits or quantitative trait loci (QTL)^11^,^12^. Day-neutrality is probably the most agriculturally desirable trait for strawberry breeding. Unlike short day (SD) strawberries that initiate flowering when day-length is short (<14 hours) and temperature is low (<15 °C)^13^, the day-neutral (DN) genotypes are photoperiod insensitive and continue to flower in the long days of spring and summer, provided that temperatures stay moderate (below 30/26 °C day/night)^14^. This provides DN cultivars commercial advantage due to their extended harvesting season^15^.

A number of markers associated with flowering response have been identified for diploid *Fragaria* species. Two SCAR markers, SCAR 1 and SCAR 3 were mapped at 3.0 cM and 1.7 cM, respectively from the *SEASONAL FLOWERING LOCUS* (*SFL*) using a seasonal diploid *F. vesca* spp. *vesca* x perpetual *F. vesca* spp. *semperflorens* testcross population, whilst SCAR 2 was inseparable from the locus^16^. Further, RAPD genotyping of F_1_ population from the everbearer ‘Ever berry’ x June bearer ‘Toyonoka’ cross allowed mapping of two RAPD markers (OPE07-1 and OPB05-1) at 11.8 cM and 15.9 cM on either side of the everbearing gene^17^. However, the practical application of these markers was limited due to weak linkages between markers and genes^18^ and differences in the inheritance of day-neutrality between diploid and octoploid strawberries^12^.

Other studies have demonstrated that the flowering habit in diploid *F. vesca* is regulated by a single dominant locus, whilst the inheritance of day-neutrality in octoploid strawberry is either a monogenic^15^,^19^ or polygenic trait^20^,^21^,^22^. In addition, several QTL with modest effect were identified by constructing a linkage map using AFLP markers with DN ‘Tribute’ x SD ‘Honeoye’ mapping population. However, the authors admitted that a major dominant gene controlling day-neutrality may have been missed due to the relatively diffuse map^13^. Using a mapping population derived from the same parents, a major QTL flanked by markers ChFaM011-163T and ChFaM148-184T was identified, in which the latter marker was strongly associated with day-neutrality and runner production^14^. This study was further supported by Gaston *et al*.^23^ who demonstrated that a single major QTL named *FaPFRU* is related to the balance of flowering and vegetative development. However, these markers have not been mapped against the *F. vesca* draft genome^24^ to identify closely linked genes possibly involved in regulating day-neutrality in strawberry. Moreover, several flowering genes previously identified in *Arabidopsis thaliana* including *PHYTOCHROME A* (*PHA*), *CONSTANS* (*CO*), *SUPPRESSOR OF OVEREXPRESSION OF CONSTANS 1* (*SOC1*) and *LEAFY* (*LFY*) also exist in *F. vesca*^25^,^26^. Yet, the specific genes controlling the critical switch from vegetative to reproductive growth remain unknown.

Bulked Segregant Analysis (BSA), developed by Michelmore *et al*.^27^ is a rapid method for detecting DNA markers linked to any specific gene in the genome without the need for inbred parents. This method involves screening two DNA bulks with contrasting phenotypic traits to identify genomic loci corresponding to the trait of interest^27^. Application of the BSA approach in conjunction with molecular markers such as random amplified polymorphic DNA (RAPD), amplified fragment length polymorphism (AFLP) and simple sequence repeats (SSR) have been widely used to understand the genetic control of agronomically important traits. For instance, discovery of markers associated with drought resistance in maize^28^, identification of QTL linked to pod and kernel traits in peanut^29^ and tagging of brown planthopper resistance genes in rice^30^. In strawberry, coupling of RAPD and AFLP markers with Bulked Segregant Analysis (BSA), have enabled identification of the *Rf l1* and *Rca2* locus that control resistance to *Phytophthora fragariae* and *Colletotrichum acutatum*, respectively. These markers have been converted to sequence characterised amplified region (SCAR) markers and used in a limited number of breeding programs^31^,^32^. In recent years, BSA has also been combined with microarray for phenotype-genotype studies. For example, application of BSA in combination with Diversity Arrays Technology (DArT) has been used to identify the DNA region linked to pubescent leaf (mPub) alleles in barley^33^. Combination of BSA with Single Feature Polymorphism (SFP) arrays and SNP arrays have also been applied to map genes associated with mutant phenotypes in *Arabidopsis*^34^,^35^,^36^,^37^ and soybean^38^ and to discover novel QTL in *Arabidopsis*^39^ and yeast^40^. These findings suggest that BSA is flexible and amenable to other marker systems.

We explored the utility of the subtracted gDNA microarray-assisted BSA for identifying molecular markers associated with day-neutrality in octoploid strawberry. This study describes the development of a strawberry-specific SDA named *Fragaria* Discovery Panel (FDP) and demonstrates its ability to detect polymorphic markers among strawberry genotypes varying in flowering strength. In addition, we also report the identification of an putative markers FaP2E11 which could possibly be developed as a molecular tool to rapidly screen strawberry seedlings for day-neutrality at a very early stage.

## Results and Discussion

### Assessment of flowering response and bulking of F_1_ segregants

We evaluated the day-neutrality strength of progeny from three segregating populations (DN ‘01-061-311’ x SD ‘Juliette’, DN ‘01-061-311’ x DN ‘05-069-63’ and DN ‘01-061-311’ x DN ‘05-069-194’. We adapted a well-developed scoring method from Shaw and Famula^15^. To ensure accurate phenotyping, we monitored the day length in Melbourne during summer and spring. Normally a short day plant will start fruiting in spring and turn vegetative completely by mid-summer. Therefore any plants that stop flowering by early January are considered as short day plants. Genotypes were considered as day-neutral if they flowered under short days of spring between September to October (11.5–12.5 h) and continued flowering under the long days of summer from December to January (14–15 h). In addition, genotypes were considered strong day-neutral if they produce flowers on runners in mid-summer under long day conditions in January. More importantly, the flowering behaviour of strong day-neutral and short day genotypes that we used in the phenotypic extreme bulks are less influenced by subtle environment variations observed in odd years.

We aim to collect a minimum of 10 plants per flowering response class for BSA as recommended by Collard *et al*.^41^. Plants with severe damage or disease symptoms were eliminated from the study. Out of the 600 F_1_ plants evaluated from all the crosses, most exhibited the SD phenotype whilst only a few exhibited varying levels of day-neutrality. 49 plants representing 10 SD, 18 weak- and 19 intermediate-DN genotypes were randomly selected from the pool. Only two plants exhibiting transgressive segregation, i.e. strong DN phenotypes were found, one from each of the two DN x DN crosses (Table 1). Instead of pooling all the DN genotypes into one bulk regardless of their flowering strength, the F_1_ plants were pooled into four predefined bulks: DN1, DN2, DN3 and SD corresponding to strong day-neutral, intermediate day-neutral, weak day-neutral and short day, respectively. This avoided homogenisation of loci controlling the day-neutrality trait due to gene dosage effect^20^,^22^. Overall, our results indicate that day-neutrality may be a recessive trait since most of the F_1_ individuals were SD plants, or the trait may be controlled by genes with varying dosage effects.

### Validation of *Fragaria* Discovery Panel (FDP)

The FDP was validated using the driver (non-angiosperm) and tester (strawberry) DNA. Only three (FaP1G12, FaP4D5, and FaP4C2) out of the 290 features hybridised with the driver target, suggesting a high (99%) subtraction efficiency. The three features may represent non-subtracted DNA fragments and were therefore removed from subsequent analysis. This subtraction efficiency is comparable to the first prototype angiosperm SDA (97.0%) and Asterids-specific SDA (99.6%)^2^,^4^. The high subtraction efficiency in this study was achieved using 1:60 tester:driver ratio as recently described for the *Echinacea*-SDA^6^ that enabled 97% efficiency compared to the *Salvia*-SDA where 1:30 tester:driver ratio produced 88% efficiency^5^. Therefore, we are confident that all common DNA sequences between strawberries and non-angiosperms were eliminated, and the resulting FDP is significantly enriched with angiosperm- and/or strawberry-specific DNA sequences.

A total 28 out of 290 features did not hybridise with the tester/driver pool. We suspect that eight out of the 28 features may be the spike-in control human skeletal muscle that was used during the SDA construction because these features did not hybridised with any of the strawberry genotypes. Interestingly, the other 20 features hybridised to the DNA of some individual strawberry genotypes (data not shown). One possible explanation is the ‘dilution effect’ reported by Jayasinghe *et al*.^2^, where DNA fragments with low frequency in the plant genome can remain undetected if the target gDNA pool consists of more than one plant genotype. These low copy number DNA sequences could represent potential cultivar or genotype-specific DNA markers, if not related to day-neutrality. For the purpose of this study, these features were retained during data analysis to avoid eliminating useful features, resulting in a set of 287 polymorphic DNA sequences on the FDP. Next, we explored the utility of the array for marker discovery.

### Identification of polymorphic features associated with day-neutrality

For this study, to reduce the chances of detecting false positives, we employed three different statistical analyses at the later stage to select the most informative features associated with day-neutrality. Firstly, we identified polymorphic features based on Discriminant Function Analysis (DFA) of hybridisation signals from DN1-SD, DN2-SD and DN3-SD comparisons. The DFA revealed a total of six variable features for each flowering response class as the best polymorphic features that could maximally separate SD from DN1, DN2 and DN3, with 100% correct grouping for the original cases in the training set (Table 2). However, the accuracy of group membership prediction for test set was slightly compromised, with only 66.7% of the new cases correctly classified into either DN2 or SD, suggesting that FaP2E2, FaP3H2, FaP3E5, FaP1E10, FaP3E7 and FaP2E9 features selected by DFA may not be the best predictor variables discriminating between SD and DN2 (Table 2). In contrast, the rate of correct classification for both, strong DN and weak DN genotypes was higher compared to the intermediate DN, where 83.3% of the new cases in the test sets were correctly predicted (Table 2). This suggested that all the six features selected by DFA for SD-DN1 and SD-DN3 comparisons were good predictor markers.

One possible explanation for a higher rate of correct classification for SD-DN1 and SD-DN3 comparisons was easy identification of strong DN and SD phenotypes in the field. For example, only plants with flowers on runners were considered as strong DN^42^, whilst plants that produced runners only but no flowers under long day and high temperature conditions were regarded as SD^43^,^44^. Comparatively, it is more difficult to classify plants with intermediate DN flowering as some of the late-fruiting DN genotypes that do not need much chilling may be classified as SD when scored in January (midsummer). Further, SD genotypes with minimum chilling requirements may also initiate flower bud formation in mild summer conditions and be misclassified as DN plants^19^. Nevertheless, our classification results are comparable to previous studies, where 65–100% correct classifications were achieved using the DFA-derived classification model to predict association between PCR-based markers and genetic diversity in wild emmer wheat^45^, agronomic traits in rice^46^, disease resistance in maize^47^,^48^ and stress tolerance index in Sardari wheat ecotypes^49^. These studies also demonstrate consistency between DFA and QTL mapping^46^,^47^,^48^, suggesting that DFA is a reliable approach to identify informative markers for agriculture applications.

Since low numbers of individuals were used in the sample bulking, we validated the DFA-selected features with Fisher’s ratio, to measure the linear discriminating power of the FDP features. Of all the DFA-selected features, FaP1A1 and FaP2E11 showed high Fisher’s ratio (Table 3), indicating a significant difference in their group means. Conversely, other DFA-selected features yielded lower Fisher’s ratios, suggesting that these features either have minimal differences between group means (low signal intensities) or larger sum of variances of two groups (greater variation between technical replicates). Similarly, 28 other features with high Fisher’s ratio (Table 3) were not selected by DFA. These discrepancies could be attributed to the different algorithms used to compute the variables in DFA and Fisher’s ratio. In DFA, the function is calculated based on weighted combination of variables where group differences on the function are maximised^50^ whilst Fisher’s ratio involves only a single variable discriminating two groups based on a simple mathematical equation^51^. The subsequent Independent Samples *t*-Test further confirmed the differences between the group means for the DFA-selected features. The results revealed that the mean differences for FaP1A1, FaP2E11 and FaP2D12 were highly significant (*p* < 0.01) while FaP2E2 and FaP2E9 was significant (*p* < 0.05) (Table 4). In contrast, the group mean for other DFA-selected features were not significantly different (Table 4).

**Figure 1:**
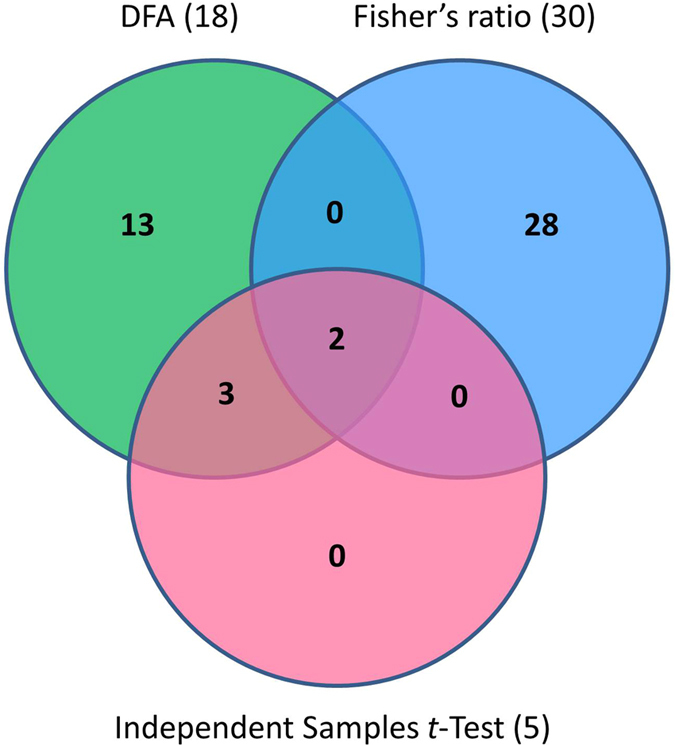
Venn diagram analysis of *Fragaria* Discovery Panel features selected by three statistical analyses. A three-way Venn diagram showing the putative DNA markers in the intersection of DFA (green), Fisher’s ratio (top 10 features; blue) and Independent Samples *t*-Test (*p* < 0.01; red) for all flowering response classes assessed.

The three-way Venn diagram revealed only two features (FaP1A1 and FaP2E11) in the intersection of the three statistical analyses (Fig. 1), where FaP1A1 and FaP2E11 are putatively associated with SD and strong DN, respectively based on the hybridisation intensities (Table 4). The intersection between DFA and Independent Samples *t*-Test revealed another three features (FaP2D12, FaP2E2 and FaP2E9) which differed significantly in their group means (Fig. 1). Their low Fisher’s ratio may be attributed to smaller magnitude of differences in group means and larger sum of the variances of two groups, as mentioned above. Thirteen features selected by DFA did not fulfil the other two statistical analyses, whilst the other 28 features that had a high Fisher’s ratio did not overlap with any other criteria, indicating that they may not be useful markers for phenotypic prediction. Nevertheless, we successfully identified two most informative markers (FaP1A1 and FaP2E11) putatively associated with day-neutrality in strawberry. Our results support the findings of previous studies, where a single major gene controlling day-neutrality in cultivated strawberry was proposed^14^,^15^,^17^,^19^. If this hypothesis is true, we do not expect a large number of markers from our study. Our results imply that perhaps only one or two loci regulate day-neutrality; however, we cannot exclude the possibility of gene dosage effect in regards to different flowering strength exhibited by the F_1_ DN genotypes.

### Sequence identity of putative DNA markers

**Figure 2:**
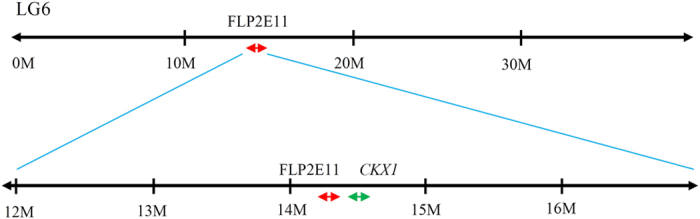
Landmark of FaP2E11 on LG6:14315954..14316207. The FaP2E11 loci and its closely linked *CYTOKININ OXIDASE 1* (*CKX1*) were found on Linkage Group 6 of *Fragaria vesca*. Red and green arrows specify the locations of FaP2E11 and *CKX1*, respectively.

Two of the most informative markers identified from the Venn diagram, FaP1A1 and FaP2E11, were sequenced to determine their identities. A similarity search against the *F. vesca* draft genome (v1.1) revealed that the full sequence of FaP1A1 matched 100% (E-value: 0.0) to a chloroplast region. Although not examined in this study, it may be worthwhile to evaluate the association of FaP1A1 locus with day-neutrality in future studies. In contrast, FaP2E11 was found to be a nuclear-specific feature and 99% of the sequence was significantly similar (E-value: 2e^−72^) to a DNA region on linkage group 6 (LG6:14315954..14316207, scf0513185:155144..155397). Four genes closely linked to FaP2E11, including *Arabidopsis thaliana* embryo defective 2765 mRNA, 6-phospogluconate dehydrogenase-like protein, cytokinin oxidase/dehydrogenase 1 (*CKX1*) and putative calcium-transporting ATPase 11 were located approximately 0.08 Mb, 0.21 Mb, 0.25 Mb and 0.38 Mb downstream of FaP2E11, respectively. Of all, *CKX1*, which is 0.25 Mb downstream of FaP2E11 (Fig. 2), is the most promising candidate associated with day-neutrality as it has been shown to be associated with regulating flowering time in *Arabidopsis*^52^,^53^.

### Proposed genetic control of day-neutrality in cultivated strawberry

In the FDP hybridisation experiments, the normalised mean SNR of FaP2E11 was three times higher in the strong DN bulk than the SD bulk (Table 4). This result suggested that the strong DN genotypes either contained more copies of the FaP2E11 putative marker or more similarity with the DNA fragment printed on the microarray. Copy number variation is often associated with diseases in human but it has recently found to be affecting traits with agronomic importance in crops^54^,^55^. Study has shown that early flowering in the allohexaploid wheat is associated with increased copy number of photoperiod response *Ppd-B1* allele. Plants with more copies of the vernalisation requirement *Vrn-A1* allele required longer periods of cold to potentiate flowering^56^. Considering strawberry as an octoploid crop, copy number variation of the FaP2E11-linked *CKX1* allele would probably be the most possible explanation for promoting the day-neutral phenotype. Therefore we propose that DN genotypes contained more copies of FaP2E11-linked *CKX1* allele printed on microarray whilst the SD genotypes contained fewer copies of this allelic sequence.

The role of cytokinin oxidase in flowering regulation was reported in several functional studies. Overexpression of *CKX* gene has been shown to suppress flowering in *Arabidopsis* plants^52^. Conversely, an insertional mutagenesis study in *Arabidopsis* showed that the *ckx3 ckx5* double mutant produced significantly more flowers with a larger inflorescence meristem compared to the wild type and the single mutants^53^. Importantly, D’Aloia *et al*.^57^ provided an initial glimpse of the role of cytokinin in regulating these flowering-time genes by using the *Arabidopsis* mutants for *CO*, *SOC1*, *FLOWERING LOCUS T* (*FT*), the *FT* paralogue *TWIN SISTER OF FT* (*TSF*), and the bZIP transcription factor FD. The study demonstrated that *tsf-1* and *soc1-2* single mutants did not respond to N^6^-benzylaminopurine (BAP) treatment, indicating that *TSF* and *SOC1* are required to initiate flowering in response to BAP. In contrast, the *ft-10* mutant continues to form flower buds, suggesting that endogenous cytokinin is associated with a different flowering route in *Arabidopsis* which bypasses *FT* but requires its paralogue *TSF*^57^. These studies implied that cytokinins are required for floral induction, and cytokinin oxidase is a negative regulator for flowering.

The current knowledge of flowering regulation in *Arabidopsis* indicates that accumulation of CO protein as day length increases results in flower initiation. This is related to the fact that CO protein is stabilised by light and degraded in darkness^58^. Surprisingly, no accumulation of CO protein was observed in some of the day-neutral strawberry lines tested in a previous study^59^, suggesting that flowering in strong DN genotypes may be regulated by an alternative flowering mechanism^60^. Based on our current results, we propose that strong DN genotypes contain more copies of the FaP2E11 linked to the low enzyme activity *CKX1* allele, allowing accumulation of cytokinin in the leaves and shoot apical meristem (SAM) under unfavourable flowering condition (long day) and thereby promoting flowering in summer^61^. In contrast, SD genotypes may contain fewer copies of this allelic variant but more copies of wild type *CKX1* allele. Consequently, cytokinin degradation occurs at a faster rate, hence flower initiation is repressed.

**Figure 3:**
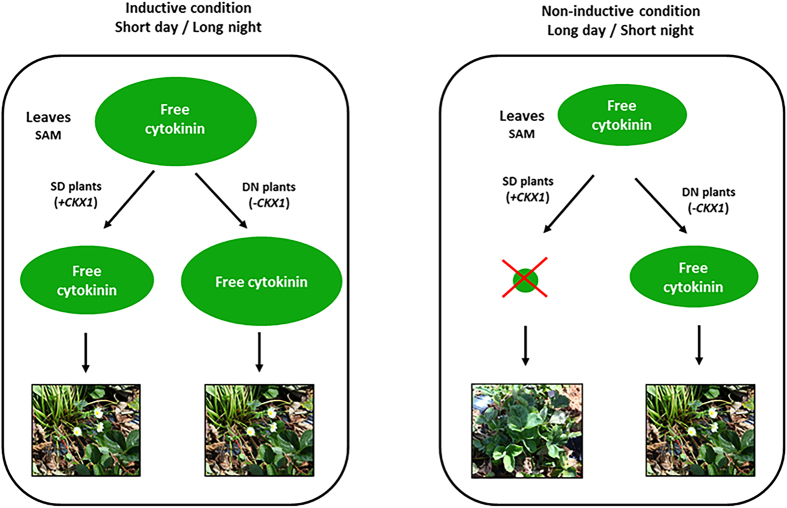
Proposed model of flowering response controlled by the copy number of the allelic variants of *CKX1* allele in SD and DN strawberry genotypes. SAM: Shoot apical meristem. +*CKX1*: Wild type allele;- *CKX1*: Low enzyme activity allele.

Based on this hypothesis, we propose a model to explain flower induction in both SD and DN genotypes under favourable and unfavourable conditions (Fig. 3). When the day is shorter than night (spring), higher levels of free cytokinins accumulate in the leaves and shoot apical meristem (SAM)^62^. Although the SD plants contain more copies of wild type *CKX1* allele, the degradation of cytokinins is slower as *CKX* activity is promoted by light^63^. As DN plants contain more copies of the low enzyme activity *CKX1* allele, the levels of free cytokinins remain the same. Therefore, flowering is induced in both SD and DN strawberry genotypes. In contrast, when the day is longer than night (summer), lower levels of free cytokinins accumulate in the leaves and SAM^62^. Due to more copies of wild type *CKX1* allele present in the SD plants, free cytokinins are degraded quickly in the plants, thus suppressing flowering and causing runners to be vigorously produced. Conversely, since the DN plants contain more copies of low enzyme activity *CKX1* allele, the levels of free cytokinins in leaves and SAM are still sufficient to promote flowering. Therefore, only the DN genotypes continue to flower in summer but produce fewer runners compared to the SD genotypes. Our proposed model is in agreement with Gaston *et al*.^23^, where the allelic variants of a major QTL, named *FaPFRU* located on LGIVb-f regulates the balance between sexual and asexual reproduction in cultivated strawberry. The authors suggested that the ‘wild-type’ allele of *FaPFRU* controls seasonal flowering whereas the ‘variant’ allele leads to perpetual flowering^23^. It would be interesting to investigate whether there is any cytokinin oxidase gene closely linked to *FaPFRU*. Our explanation is also consistent with previous studies, where cytokinin application suppressed runnering in SD strawberries^64^ and flowering inhibits runner formation in both SD and DN genotypes^43^.

## Conclusions

To the authors’ knowledge, this is the first report on the applicability of the subtracted gDNA microarray in conjunction with BSA for marker discovery in commercial polyploid crop species. We successfully identified a putative DNA marker, FaP2E11 highly associated with day-neutrality trait in octoploid strawberries. It is important to determine the copy number of FaP2E11-linked *CKX1* allele in both SD and DN genotypes used in this study. We hypothesise that copy number differences of the FaP2E11-linked *CKX1* variants could be the possible cause of different flowering strength between SD and DN genotypes.

## Methodology

### Plant materials and gDNA extraction

In order to construct the subtracted gDNA library, five strawberry genotypes and nine non-angiosperm species were sourced (Table 5). The strawberry genotypes selected as tester were an American (Albion) and Australian (Juliette) cultivars, and three promising breeding lines carrying genetic materials from the European (07-102-41 and 07-095-35) and Japanese (04-069-91) strawberries. This selection of genotypes ensured the inclusion of a wide range of genetic background to increase the discriminatory power of the array technology^3^. Leaf tissues of the strawberry genotypes were collected from a strawberry farm in Coldstream and Wandin North, Victoria. To selectively capture flowering related fragments in strawberries, four ferns, three coniferous trees, one cycad and one ginkgo were used as driver for subtraction against the tester pool. All the non-angiosperms were obtained from accredited nurseries and from the Chinese Herbal Garden, RMIT Bundoora Campus.

Additionally, three segregating populations: (1) DN ‘01-061-311’ x SD ‘Juliette’, (2) DN ‘01-061-311’ x DN ‘05-069-63’ and (3) DN ‘01-061-311’ x DN ‘05-069-194’ with a population size of 200 were chosen for day-neutrality assessment. Juliette is an Australian short-day cultivar highly preferred by consumers and has been used as a parent in many crosses in the Australian strawberry breeding program. In contrast, 01-061-311, 05-069-63 and 05-069-194 are three strong day-neutral advance breeding lines bred by the Victorian-based Southern Node Breeding Program and may have potential for commercial release^65^. For each segregating population, both parental genotypes and selected F_1_ progenies expressing a variety of flowering responses were harvested for BSA.

Genomic DNA of all plant materials was extracted using Qiagen^TM^ DNeasy^®^ Plant Mini Kit (Qiagen, Valencia, CA) according to manufacturer’s guidelines. DNA concentration and purity was evaluated spectrophotometrically whilst the integrity was determined using 1.5% agarose gel electrophoresis.

### Assessment of day-neutrality and Bulked Segregant Analysis (BSA)

Day-neutrality was assessed by scoring the individual F_1_ progeny plants obtained from the three segregating populations for the presence of flowers in midsummer (05/01/2012 and 10/01/2012) according to the subjective scoring method described by Shaw and Famula^15^ with a few modifications. Plants were assigned into four classes based on a scale from 1 to 4 as follows: 1 = flowering on runners and two or more recently emerged inflorescences on the mother plant, 2 = two or more recently emerged inflorescences, 3 = at least one recently emerged inflorescences and 4 = no flowers or fruits (Table 6) Plants.

Subsequently, BSA was performed by pooling equal quantities of DNA from F_1_ individuals into four different bulks defined as ‘strong day-neutral (DN1)’, ‘intermediate day-neutral (DN2)’, ‘weak day-neutral (DN3)’ and ‘short-day (SD)’ bulks (Table 6) to a final amount of 2 μg. The number of individuals in each bulk ranged from 2 to 19 plants depending on the flowering response class. Instead of classical BSA where the bulks were pre-screened with primers, these bulks were used as probes for hybridisation with the *Fragaria* Discovery Panel (FDP).

### Genomic DNA subtraction and FDP construction

**Figure 4:**
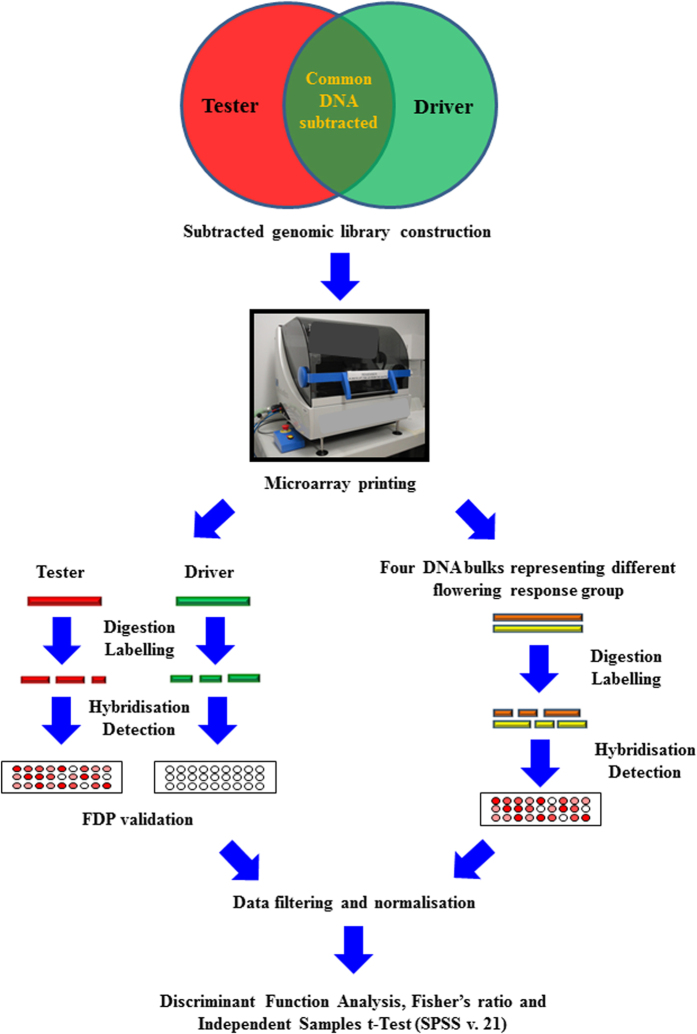
Experimental workflow of *Fragaria* Diversity Panel (FDP) construction and its application on marker discovery.

The experimental workflow of FDP construction and hybridisation with DNA bulks is summarised in Fig. 4. DNA subtraction and microarray construction were performed as described by Jayasinghe *et al*.^2^ with a few modifications. Firstly, genomic representations were prepared by pooling equal quantities of DNA extracted from five strawberry genotypes and nine non-angiosperm species (Table 5) into tester and driver pools, respectively to a final amount of 4 μg and fragmented overnight with 5 units of *Alu*I and *Hae*III (NEB, Ipswich, MA) in a 100 μL digestion mixture. DNA subtraction was performed using PCR-Select^TM^ cDNA Subtraction kit (Clontech, Mountain View, CA) according to the manufacturer’s instructions but the tester:driver ratio was increased to 1:60^6^. Microarray probe preparation and printing was performed according to Jayasinghe *et al*.^2^ except that an aromatase gene was added as a spike-in control to normalise systematic variation across slides because it derived from the ovary of Murray River rainbowfish (*Melanotaenia fluviatilis*)^66^ and therefore not expected to cross-hybridise with any sequences in the strawberry genome. A total of 290 subtracted clones together with six positive controls, nine negative controls, two printing controls and one spike-in control were used to construct a 308-feature FDP. Two subarrays, each with six technical replicates (each technical replicate consisted of 308 samples), were arrayed onto Corning^®^ GAPS^TM^ II coated slides (Corning Incorporated, NY, USA) using a BioRobotics^®^ MicroGrid II Compact array printing robot (Genomics Solutions, Ann Arbor, MI).

### Target labelling and hybridisation

The FDP was firstly validated by individually hybridising tester and driver pools onto the array to evaluate the subtraction efficiency of the SSH process. Secondly, the FDP was evaluated for its ability for marker discovery by hybridising the four DNA bulks representing different flowering response onto the FDP. In all cases, target labelling involved double digestion of 2 μg of DNA with *Alu*I and *Hae*III and purification with QIAquick PCR Purification kit (Qiagen, Valencia, CA). Approximately 200 ng of purified digested target DNA was then labelled with Biotin-11-dUTP molecules for 20 hours using Biotin DecaLabel^TM^ DNA Labelling kit (Fermentas, Pittsburgh, PA) according to the manufacturer’s guidelines. Hybridisation of the biotinylated DNA targets onto the FDP and fluorescent detection using a biotin-streptavidin system was performed as described by Mantri *et al*.^4^. All hybridisations were performed with six technical replicates and two biological replicates to ensure microarray reproducibility, producing a total of 12 data points per feature for subsequent statistical analysis. All microarray experiments were compliant with MIAME guidelines and all data have been deposited in Gene Expression Omnibus (GSE70145).

### Array scanning and data analysis

The FDP slides were scanned at 10 μm resolution with 55% of PMT gain at 633 nm (Cy-5, red laser) to reduce the background noise using the ScanArray G_x_ Microarray Scanner (PerkinElmer, USA). The images were captured and quantified using PerkinElmer ScanArray Express^®^ software v 4.0. The signal intensity of each spot was quantified using the adaptive circle method and normalised using the LOWESS function. The quality and status of each feature was checked and flagged manually, allowing for the elimination of empty features (negative controls and unbound samples) and bad features (contaminated features or features with high background noise). Signal-to-noise ratio (SNR) was obtained for each feature as it was considered to have the most accurate background correction. All spots with a SNR greater than 7 in more than half of the technical replicates were considered as good features^5^. The quantified FDP data was exported to Microsoft Excel. A total of 287 good features that passed all the quality control criteria were used for subsequent data analysis, including (1) normalisation between technical replicates and hybridisations using spike-in control normalisation method, (2) calculation of mean for the normalised SNR of each feature between technical replicates and (3) combination of the biological replicates to produce a fingerprint comprising one value per feature.

### Statistical analysis

The FDP data was subjected to Discriminant Function Analysis (DFA) to identify molecular markers associated with day-neutrality. The four predefined groups (DN1, DN2, DN3 and SD) and the normalised mean SNR of the 287 FDP features were used as dependent and independent variables, respectively. DFA was performed with stepwise method (IBM SPSS Statistics v. 21) to select a set of features that best discriminate between the two phenotypic groups. Wilks’ lambda was used as the selection criterion to determine the classification efficiency of each feature based on the default *F* probability values (Entry = 0.05, Removal = 0.10). The selected features were then employed to construct and validate a discriminant function for each group using Fisher’s classification function coefficients, and tested their ability to classify new cases into the correct phenotypic group. In this study, six technical replicates (original cases) from the first biological replicate of a given phenotypic group were assigned as training set to predict the group membership of the other six technical replicates (the new cases) from the second biological replicate, which is the test set. A reciprocal analysis was also performed and the performance of the discriminant function was evaluated by calculating the percentage of correct classification determined from the number of misclassified cases.

To validate the DFA-selected features, Fisher’s ratio and Independent Samples *t*-Test were employed to eliminate irrelevant features. Fisher’s ratio was calculated according to Lohninger^51^:


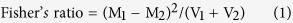


where M_1_ = Mean of the normalised SNR for each feature in the SD bulk. M_2_ = Mean of the normalised SNR for each feature in the DN bulk. V_1_ = Variance of the normalised SNR for each feature in the SD bulk. V_2_ = Variance of the normalised SNR for each feature in the DN bulk.

Independent Samples *t*-Test (IBM SPSS Statistics v. 21) was performed using six technical replicates and two biological replicates of the SD and DN bulks as variables. Only the features showing high Fisher’s ratio (top 10) and significant differences between the group means of SD and DN bulks (*p* < 0.01) were retained for further analysis. Finally, a three-way Venn diagram was generated (http://www.pangloss.com/seidel/Protocols/venn.cgi) to identify putative DNA markers that fulfilled all three selection criteria.

### DNA sequence analysis

Plasmids corresponding to the putative DNA markers were sequenced bi-directionally at Macrogen Inc. (Korea) using T7 and Sp6 primers. Similarity search was performed against the *Fragaria vesca* draft genome (v1.1) using PFR Strawberry Server (https://strawberry.plantandfood.co.nz/) and confirmed with Genome Database for Rosaceae (http://www.rosaceae.org/tools/ncbi_blast). Sequence identity with an E-value < 1e^−5^ was considered significant. All sequences have been deposited in GeneBank (KT162989 – KT163008). Subsequently, genes located within 5 centiMorgan (cM) on either side of the putative DNA markers were manually searched using PFR Strawberry Server based on previously mapped genes available in Strawberry Genbank and general RefSeq mRNA database. By assuming the genetic length of a normal chromosome as 100 cM^67^, the physical distance covering 5 cM was calculated following the equation below:


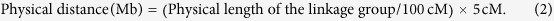


## Additional Information

**How to cite this article**: Gor, M. C. *et al*. Application of subtracted gDNA microarray-assisted Bulked Segregant Analysis for rapid discovery of molecular markers associated with day-neutrality in strawberry (*Fragaria x ananassa*). *Sci. Rep.* **6**, 32551; doi: 10.1038/srep32551 (2016).

## Figures and Tables

**Table 1:** The number of F_1_ progeny collected for each DN × SD and DN × DN crosses based on flowering strength.

Crosses	DN1	DN2	DN3	SD
DN × SD				
01-061-311 × Juliette	0	6	6	10
DN × DN				
01-061-311 × 05-069-63	1	8	7	0
01-069-311 × 05-069-194	1	5	5	0
Total	2	19	18	10

DN1: Strong day-neutral.

DN2: Intermediate day-neutral.

DN3: Weak day-neutral.

SD: Short-day.

**Table 2:** Classification results for the training set and test set based on the DFA-selected features for each flowering response group.

Floweringresponse	DFA-selected markers	Classification Results (%)	
		Training set	Test set
DN1 – SD	FaP2E11, FaP3F4, FaP2D6, FaP2B4, FaP2D12, FaP2G4	100.0	83.3
DN2 – SD	FaP2E2, FaP3H2, FaP3E5, FaP1E10, FaP3E7, FaP2E9	100.0	66.7
DN3 – SD	FaP1A1, FaP2A2, FaP3D4, FaP3B3, FaP3B10, FaP2E9	100.0	83.3

**Table 3:** List of the top 10 features ranked in decreasing order based on their respective Fisher’s ratio for the flowering response groups.

Fisher’s ratio	Flowering response group		
Ranking	DN1–SD	DN2–SD	DN3–SD
1	FaP1G3	FaP1A1	FaP1A1*
2	FaP2G5	FaP4A8	FaP2G12
3	FaP2E11*	FaP2D3	FaP3F7
4	FaP1B10	FaP4D1	FaP2G11
5	FaP3F7	FaP2F11	FaP3A5
6	FaP1B9	FaP2G5	FaP4B1
7	FaP2E4	FaP1G7	FaP2D3
8	FaP1G1	FaP3B1	FaP1C8
9	FaP1C4	FaP2C11	FaP2F5
10	FaP1H8	FaP3F7	FaP3G6

^*^Putative DNA markers which were also selected by DFA.

**Table 4:** The results of group statistics and independent samples *t*-test for the putative DNA markers selected by DFA.

Flowering response group	DFA-selected markers	Day-neutral		Short-day		*t*-test (for two bulks)		
		Mean^b^	*s*	Mean^b^	*s*	*t* value	*df*	*p*
DN1 – SD	FaP2E11^a^	743.53	252.08	259.98	148.78	5.723	22	**
	FaP3F4	133.90	71.34	210.06	191.96	1.288	22	ns
	FaP2D6	188.96	119.83	337.52	250.68	1.852	22	ns
	FaP2B4	74.95	53.71	117.72	89.99	1.414	22	ns
	FaP2D12	223.23	146.47	433.96	143.61	−3.559	22	**
	FaP2G4	161.45	69.35	105.77	65.22	−2.026	22	ns
DN2 – SD	FaP2E2	85.71	48.94	232.53	172.45	−2.837	12.8	*
	FaP3H2	545.45	243.59	368.65	322.27	−1.516	22	ns
	FaP3E5	104.54	41.97	131.37	108.95	0.796	22	ns
	FaP1E10	200.32	118.04	179.75	116.79	−0.429	22	ns
	FaP3E7	202.05	85.17	296.19	232.35	1.318	22	ns
	FaP2E9	40.82	25.66	67.17	30.59	2.287	22	*
DN3 – SD	FaP1A1^a^	0.27	0.35	713.71	499.52	−4.948	11.0	**
	FaP2A2	253.29	117.27	354.78	287.26	1.133	14.6	ns
	FaP3D4	133.98	82.77	137.14	70.63	0.101	22	ns
	FaP3B3	227.30	136.36	300.66	237.70	−0.927	22	ns
	FaP3B10	38.87	18.90	49.46	23.85	1.206	22	ns
	FaP2E9	46.73	19.48	67.17	30.59	1.953	22	ns

*s*: Standard deviation. **Significant at *p* < 0.01. *Significant at *p* < 0.05. ns Not significant.

^a^Putative DNA markers which displayed high Fisher’s ratio values.

^b^Mean signal-to-noise ratio (SNR) of two biological replicates and six technical replicates.

**Table 5:** Strawberry genotypes and non-angiosperms used in the subtracted library construction.

Plant Materials			Sources
Strawberry Genotypes	Australian	Juliette	Coldstream
	American	Albion	Coldstream
	Breeding lines	07-102-4107-095-3504-069-91	Wandin NorthWandin NorthWandin North
Non-angiosperms	Ferns	*Dryopteris kurataeDicksonia antarcticaAsplenium australasicumBlechnum tabulare*	Bunnings WarehouseRMIT BundooraBunnings WarehouseBunnings Warehouse
	Conifers	*Wollemia nobilisCupressus macrocarpaJuniperus communis*	RMIT BundooraBunnings WarehouseMedicinal Plant Herbarium,Southern Cross University
	Cycad	*Cycas revoluta*	Bunnings Warehouse
	Ginkgo	*Ginkgo biloba*	Digger’s Club

**Table 6:** Flowering response on a 1–4 scale based on day-neutrality strength [15].

Scale	Description of flowering response	Strength of day-neutrality
1	Flower formation on runners	Strong day-neutral (DN1)
2	2 or more recently emerged inflorescences	Intermediate day-neutral (DN2)
3	Less than 2 recently emerged inflorescences	Weak day-neutral (DN3)
4	No flowers or fruits	Short-day (SD)
